# HIV–tuberculosis-associated immune reconstitution inflammatory syndrome is characterized by Toll-like receptor and inflammasome signalling

**DOI:** 10.1038/ncomms9451

**Published:** 2015-09-24

**Authors:** Rachel P. J. Lai, Graeme Meintjes, Katalin A. Wilkinson, Christine M. Graham, Suzaan Marais, Helen Van der Plas, Armin Deffur, Charlotte Schutz, Chloe Bloom, Indira Munagala, Esperanza Anguiano, Rene Goliath, Gary Maartens, Jacques Banchereau, Damien Chaussabel, Anne O’Garra, Robert J. Wilkinson

**Affiliations:** 1 The Francis Crick Institute Mill Hill Laboratory, London NW7 1AA, UK; 2 Clinical Infectious Diseases Research Initiative, Institute of Infectious Disease and Molecular Medicine and Department of Medicine, University of Cape Town, Anzio Road, Observatory 7925, South Africa; 3 Department of Medicine, Imperial College London, London W2 1PG, UK; 4 Baylor Institute for Immunology Research, Dallas, Texas 75204, USA; 5 Systems Immunology, Benaroya Research Institute, Seattle, Washington 98101, USA; 6 Department of Medicine, National Heart and Lung Institute, Imperial College London, London W2 1PG, UK

## Abstract

Patients with HIV-associated tuberculosis (TB) initiating antiretroviral therapy (ART) may develop immune reconstitution inflammatory syndrome (TB-IRIS). No biomarkers for TB-IRIS have been identified and the underlying mechanisms are unclear. Here we perform transcriptomic profiling of the blood samples of patients with HIV-associated TB. We identify differentially abundant transcripts as early as week 0.5 post ART initiation that predict downstream activation of proinflammatory cytokines in patients who progress to TB-IRIS. At the characteristic time of TB-IRIS onset (week 2), the signature is characterized by over-representation of innate immune mediators including TLR signalling and TREM-1 activation of the inflammasome. In keeping with the transcriptional data, concentrations of plasma cytokines and caspase-1/5 are elevated in TB-IRIS. Inhibition of MyD88 adaptor and group 1 caspases reduces secretion of cytokines including IL-1 in TB-IRIS patients. These data provide insight on the pathogenesis of TB-IRIS and may assist the development of specific therapies.

## Abstract (web summary)

Some patients with HIV-associated tuberculosis develop an immune reconstitution inflammatory syndrome (TB-IRIS) in response to antiretroviral therapy. Here the authors identify genes differentially expressed in patients likely to progress to TB-IRIS and find activation of Toll-like receptor and inflammasome pathways.

## Graphical abstract

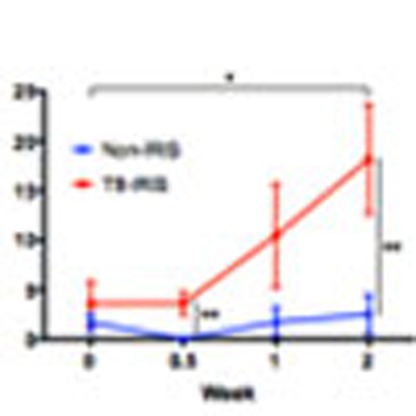

The World Health Organization (WHO) estimates that approximately one-third of the world’s population is infected with *Mycobacterium tuberculosis* (MTB), 5%–10% of whom will develop active disease^1^ with human immunodeficiency virus type 1 (HIV-1) infection being the greatest recognized risk factor for disease^2^. Seventy-eight per cent of those with HIV-associated tuberculosis (TB) live in Africa^1^. Increased access to combination antiretroviral therapy (ART) has significantly improved the clinical outcome of such patients in resource-limited settings^3^,^4^. However, up to 54% of patients develop hyperinflammatory reactions known as immune reconstitution inflammatory syndrome (IRIS) within the first month of ART initiation^5^. Paradoxical TB-IRIS is directed towards MTB antigens and is characterized by recurrent, new or worsening symptoms and signs of treated TB.

Three clinical risk factors for HIV-associated paradoxical TB-IRIS are recognized: (i) low baseline CD4^+^ T-cell count (<50–100 cells per mm^3^) before ART^6^,^7^,^8^; (ii) a short time interval between commencing TB treatment and ART^6^,^7^,^8^; and (iii) dissemination of TB to extrapulmonary sites, possibly reflecting higher bacterial load^9^,^10^. Nevertheless, the immunopathological basis of the syndrome remains incompletely understood and there is no biomarker to predict which patients will develop IRIS. Studies to date have suggested that both innate and adaptive immunity are involved, leading to hypercytokinemia and severe inflammation^11^.

Although hyperinflammation in TB-IRIS is associated with the expansion of TB antigen-specific interferon-γ (IFN-γ) producing peripheral T-helper 1 cells^12^, the absence of such expansion in some TB-IRIS cases and the presence in similar patients who do not develop the syndrome suggests that the association might not be causal^13^. Several studies have suggested that innate immunity also contributes to IRIS. In post-mortem staining of lung tissue sections from a TB-IRIS patient, most of the inflammatory cells were identified to be CD68^+^ macrophages^14^. Increased natural killer cell activation and degranulation activity^15^,^16^, and elevated neutrophil counts in the cerebrospinal fluid^17^,^18^ of tuberculous meningitis-IRIS cases have also been reported. The levels of interleukin (IL)-6 and C-reactive protein are elevated at the time of IRIS in both *Mycobacterium avium*-associated IRIS^19^ and paradoxical TB-IRIS^5^,^13^,^20^. TB-IRIS patients were also reported to have higher expression level of pattern recognition receptors (PRRs) such as Toll-like receptor 2 (TLR2)^15^,^21^,^22^,^23^. Microarray analysis of monocytes isolated from HIV-TB co-infected patients suggested an imbalance between the complement effector C1q and the inhibitor C1-INH in those who developed TB-IRIS^24^. Notwithstanding these extensive research efforts, the underlying mechanisms mediating TB-IRIS remain to be fully elucidated.

The application of systems immunology has significantly increased understanding of many diseases. In the last few years, transcriptional profiling of blood from TB patients has led to the finding of a type I IFN-inducible neutrophil-driven signature of active TB disease^25^. In this study, we used a similar unbiased whole-blood transcriptomic profiling of HIV-TB co-infected patients commencing ART, to better understand the immune pathways resulting in TB-IRIS. Patients were tracked longitudinally to ascertain development of TB-IRIS. We characterized plasma cytokine profiles, which were in keeping with the transcriptomic signature. We further demonstrated the dependence of elevated levels of cytokine on TLR signalling pathways, as well as caspase-1-dependent canonical and caspase-5-dependent non-canonical inflammasomes.

## Results

### Patient characteristics

Thirty-two patients with HIV-associated TB were included for microarray analysis. Patients in both TB-IRIS (*n*=17) and non-IRIS (*n*=15) groups had similar clinical variables, including age, sex, CD4 count and HIV viral load, and site of MTB infection (Supplementary Table 1). The majority of patients had both pulmonary and extrapulmonary TB (PTB and EPTB); the latter usually manifest as visceral TB, peripheral adenopathy and pleural effusion, with only a few cases of tuberculous meningitis (*n*=7). Patients who developed TB-IRIS had symptom onset at a median of 13 days (interquartile range (IQR): 7–21 days) after ART initiation. An additional 31 patients were not included in the microarray analysis, owing to lack of whole-blood samples, but had plasma cytokines analysed. No significant differences were observed compared with the microarray group with regard to clinical variables (Supplementary Table 1).

### Differentially abundant transcripts at 2–5 days post ART

**Figure 1:**
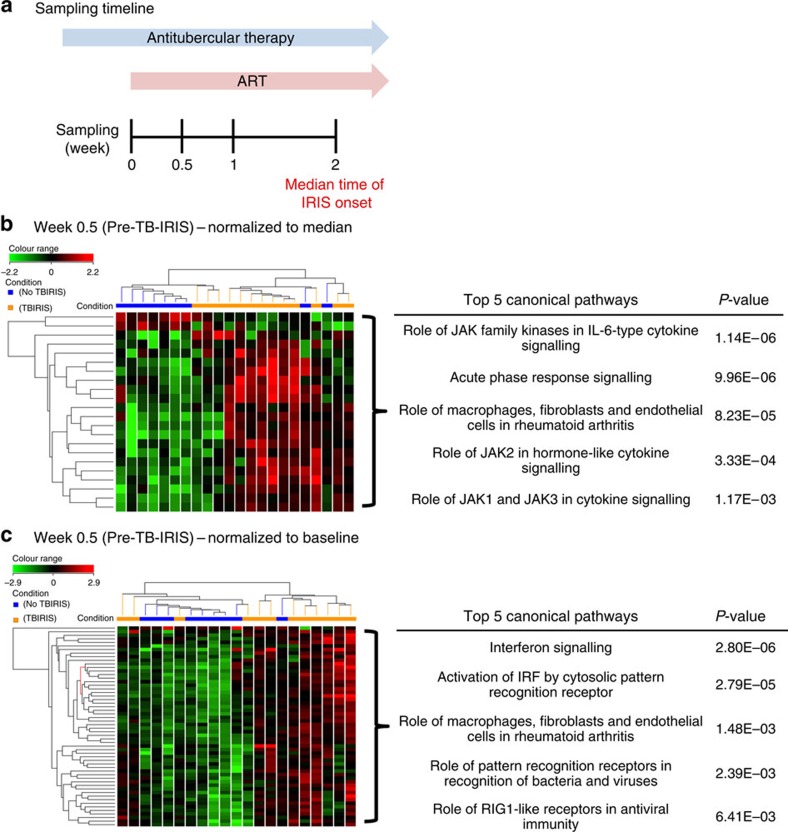
Differentially abundant transcripts associated with progression to TB-IRIS were identified early before onset of clinical symptoms. (**a**) Whole-blood samples were collected longitudinally and the median onset of TB-IRIS was 13 days. (**b**) Microarray was performed with 22 whole-blood samples (13 TB-IRIS and 9 non-IRIS). Twenty-two transcripts (20 genes) were differentially abundant at week 0.5 (days 2–5 post ART) in those patients who eventually developed TB-IRIS, compared with non-IRIS. Transcript intensity values were normalized to the median of all samples. Transcripts are clustered vertically where green represents decreased abundance and red represents increased abundance. Patient arms are clustered horizontally where blue represents non-IRIS and orange represent TB-IRIS. Significance of pathways over-represented by differentially abundant genes was calculated by Fisher’s exact test corrected for Benjamini–Hochberg false discovery rate (BH-FDR) (*P*=0.05). (**c**) Using an alternative analytical approach by normalizing transcript intensity to its corresponding baseline (week 0) value, 55 transcripts (50 genes) were found to be differentially abundant in TB-IRIS patients, compared with non-IRIS controls. Significance of pathways over-represented by differentially abundant genes was calculated by Fisher’s exact test corrected for BH-FDR (*P*=0.05). One sample was excluded from the analysis, owing to the lack of corresponding baseline.

Longitudinal whole-blood samples were collected from patients with HIV-associated TB commencing ART (Fig. 1a). We investigated the molecular path of immune dysregulation leading to TB-IRIS by inspecting transcriptomic changes in TB-IRIS and non-IRIS patients by using two different normalization approaches. First was a modified method from one previously described^25^,^26^, in which signal intensities were normalized to the median of all samples. The second approach normalized each sample to its corresponding baseline (week 0). Differentially expressed genes identified by both approaches are listed in Supplementary Table 2.

At week 0, there was no transcriptomic difference between patients who did or did not develop TB-IRIS; however, within 2–5 days post ART (week 0.5) we identified 22 differentially abundant transcripts (20 genes) in patients who developed TB-IRIS (Fig. 1b) by using the normalization to median method. Functional analysis of these 22 differentially abundant transcripts indicated over-representation of the JAK/STAT signalling pathways mediated by the IL-6 cytokine family and a role for macrophages and complement (Fig. 1b). Using the alternative normalization to baseline approach, we further identified 56 differentially abundant transcripts (50 genes) in TB-IRIS patients early during ART (week 0.5) (Fig. 1c). Innate immune pathways remained dominant in TB-IRIS, represented by upregulated type I and II IFN signalling, activation of PRRs and the role of macrophages (Fig. 1c), although only 5 of the 50 genes overlapped with the 20 genes identified using the normalization to median approach. Together, the data suggest early activation of a predominantly innate inflammatory response at transcriptomic level in TB-IRIS before the onset of clinical symptoms.

### Transcriptomic signature at the time of symptom onset

**Figure 2:**
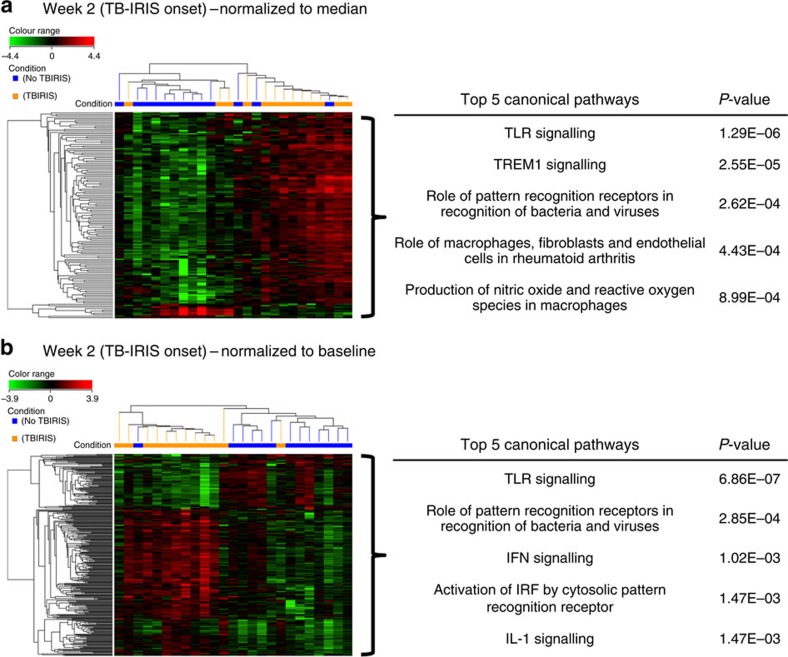
Differentially abundant transcripts in TB-IRIS onset are associated with innate signalling pathways. (**a**) Microarray was performed with 26 whole-blood samples (13 TB-IRIS and 13 non-IRIS). One hundred and thirty-eight transcripts (125 genes) were differentially abundant in the whole blood of the TB-IRIS patients at week 2. Transcript intensity values were normalized to the median of all samples. Transcripts are clustered vertically where green represents decreased abundance and red represents increased abundance. Patient arms are clustered horizontally where blue represents non-IRIS and orange represent TB-IRIS. Significance of pathways over-represented by differentially abundant genes was calculated by Fisher’s exact test corrected for Benjamini–Hochberg false discovery rate (BH-FDR) (*P*=0.05). (**b**) Using an alternative analytical approach by normalizing transcript intensity to its corresponding baseline (week 0) value, 319 transcripts (281 genes) were found to be differentially abundant in TB-IRIS patients. Significance of pathways over-represented by differentially abundant genes was calculated by Fisher’s exact test corrected for BH-FDR (*P*=0.05). One sample was excluded from the analysis, owing to the lack of corresponding baseline.

Applying the same analytical approaches described above, we identified a 138-transcript (125 gene) signature associated with TB-IRIS at week 2 by normalizing to the median (Fig. 2a and Supplementary Table 3). Functional analysis of these 125 differentially abundant transcripts indicated upregulation of several innate response pathways, with TLR and TREM1-induced inflammasome signalling being most over-represented (Fig. 2a), again suggesting an important role of innate antigen recognition and cytokine signalling in the inflammatory response in TB-IRIS. When the transcriptional data were normalized to their corresponding baseline values, we identified 319 transcripts (281 genes) differentially abundant in TB-IRIS patients at week 2 (Fig. 2b and Supplementary Table 4). Functional pathway analysis again indicated innate immune signalling pathways including TLR, IFN and IL-1 signalling to be over-represented in TB-IRIS (Fig. 2b). Of the 281 genes identified, 70 overlapped with the 125 genes identified by normalization to the median. The large number of overlapping genes indicates they are conserved in TB-IRIS regardless of analytical method. Functional analysis of the overlapping transcripts again indicated the innate immune components, with TLR, TREM-1 and IL-1 being the most enriched biological processes. Corticosteroid therapy was prescribed in ten of the patients (four of whom had microarray performed) at the time of ART commencement but it was not a confounding factor in the transcriptomic analysis.

**Figure 3:**
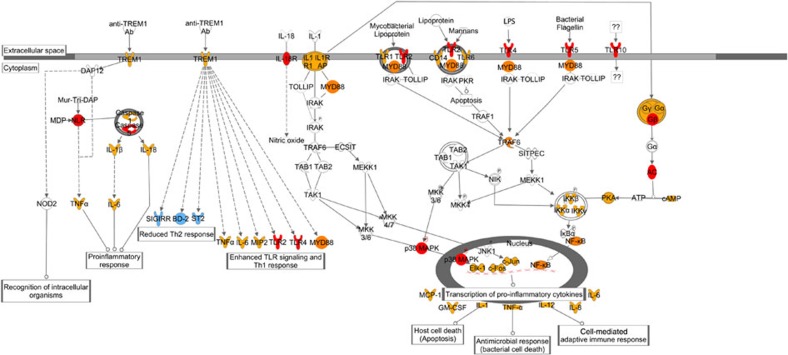
Transcriptomic signature of TB-IRIS at week 2 predicts activation of innate signalling pathways and production of proinflammatory cytokines and chemokines. The TREM1, TLR and IL-1 signalling pathways are interconnected. Molecules in red were those found to be differentially abundant in the signatures of TB-IRIS patients at week 2 (normalization to median). Molecule activity prediction was performed using Ingenuity Pathway Analysis: molecules in orange were predicted to be upregulated and those in blue predicted to be downregulated.

We next performed both regulator and molecule activity prediction^27^ of the week 2 signature by using the 125 genes identified by the normalization to median approach. Proinflammatory cytokines including oncostatin M, IFN-γ, tumour necrosis factor (TNF) and IL-1 were predicted to be the upstream regulators of the week 2 signature (Supplementary Table 5). In addition, CSF-3 (granulocyte colony-stimulating factor) and IL-5 were also predicted to be activated upstream at week 2, suggesting activation of adaptive immunity. An array of proinflammatory cytokines and chemokines were predicted to be activated at week 2, including TNF-α, IL-1β, IL-6, IL-8, IL-12, IL-18, granulocyte–macrophage colony-stimulating factor, monocyte chemoattractant protein-1 and macrophage inflammatory protein-2 (Fig. 3). Signalling appeared to be mediated by TLR and IL-1/18 receptor via the MyD88 adaptor and TREM1-induced activation of inflammasomes and TLR, resulting in the activation of nuclear factor-κB and subsequent production of cytokines and chemokines (Fig. 3).

### Plasma cytokine measurement

**Figure 4:**
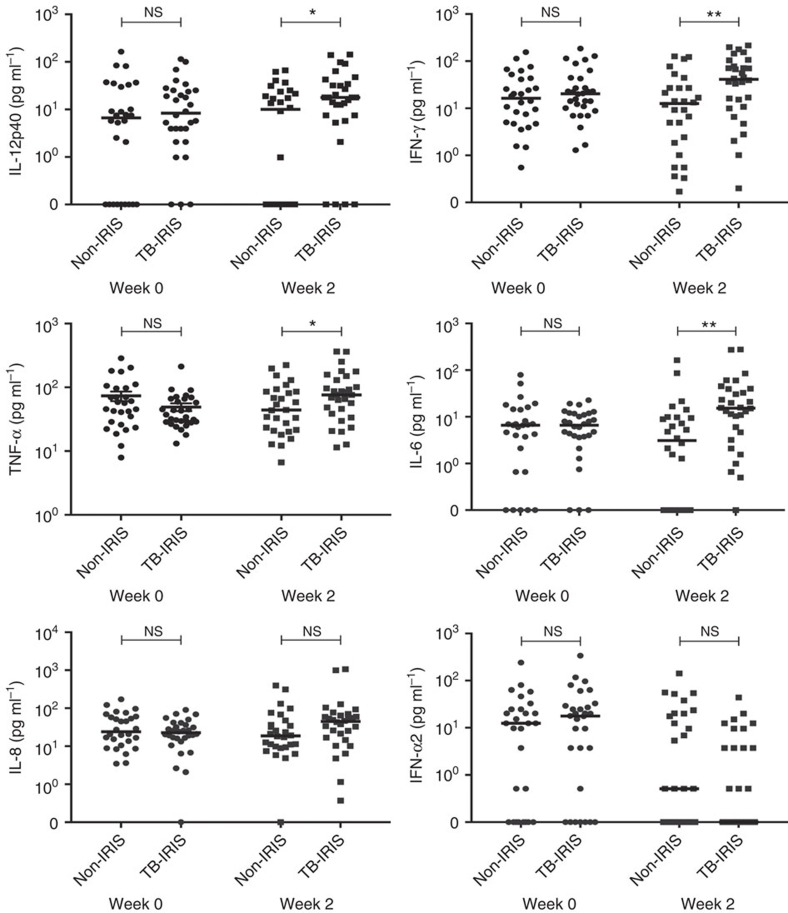
Cytokine production at week 2 of TB-IRIS reflects the transcriptomic prediction. Plasma cytokine concentrations in TB-IRIS and non-IRIS patients at week 0 and week 2 were measured. Results were analysed by Mann–Whitney *U*-test between non-IRIS and TB-IRIS at each time point. The median value of each group was shown and *P*-value was designated: *P≤0.05, **P≤0.01.

As the transcriptomic signatures predicted activation of many proinflammatory cytokines (Fig. 3), we measured the concentration of these analytes in patient plasma at both week 0 and week 2, to assess whether the transcriptomic prediction was reflected in the blood protein. Congruent with the transcriptomic signature prediction of week 2, we detected significantly higher concentrations of IL-12p40, IFN-γ, TNF-α and IL-6 in TB-IRIS patients at week 2 (Fig. 4), indicating a circulating inflammatory response at the time when clinical symptoms were observed. Our data agree with those previously reported^28^, further indicating that TB-IRIS is strongly associated with dysregulated cytokine and chemokine production. Although IL-8 and type I IFN were also predicted to be elevated at week 2, we did not detect any differences in plasma proteins between non-IRIS and TB-IRIS patients (Fig. 4).

### Validation by technically independent NanoString method

A technically independent method, the NanoString (nCounter) system, was used to further validate the 125 differentially expressed genes identified at the median time of TB-IRIS onset (week 2) by the standard normalization to median microarray analytical approach (Fig. 2a). The 125 genes contained 70 genes that were also identified using the alternative normalization to baseline approach described above (Fig. 2b). Probes to sequences differing from those detected by microarray were designed, with one being excluded from the assay because of nonspecific complementarity to other genes. Owing to insufficient quantity of RNA, eight samples (five TB-IRIS and three non-IRIS) were excluded from the NanoString assay. Seventy-six of the 124 probes that were statistically significantly different in microarray were also found significant by NanoString (Supplementary Table 6). The remaining 48 genes, although not statistically significantly different between the TB-IRIS and non-IRIS groups, also showed the same trends in abundance as the microarray. The most over-represented functional pathways of the 76 genes that were significantly upregulated in the TB-IRIS group were again from the innate immune pathways: TLR, TREM1 and IL-1 signalling.

### A 43-transcript signature that characterizes TB-IRIS

**Figure 5:**
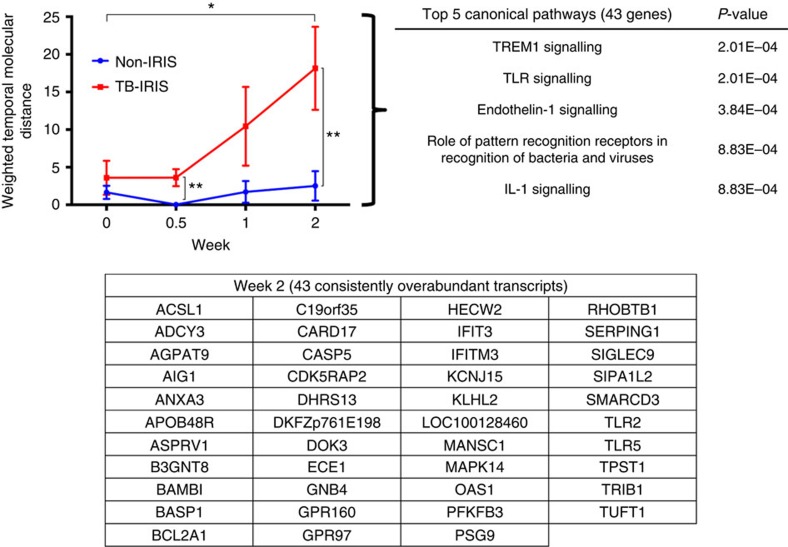
Weighted temporal molecular distance to health of 43 consistently overabundant transcripts differentiate TB-IRIS patients from non-IRIS controls. Forty-three genes were consistently overabundant in TB-IRIS by different analytical and technical platforms. These genes are associated with innate signalling pathways, namely TREM1, TLR and IL-1 signalling (Fisher’s exact test with Benjamini–Hochberg false discovery rate (BH-FDR), *P*=0.05). The weighted temporal molecular distance measured the total transcriptional perturbation of the 43 genes at each time point relative to its baseline (week 0) mean. The mean, s.e.m. and *P*-values are displayed (unpaired *t*-test with Welch’s correction; *P≤0.05 and **P≤0.01).

To determine a signature that consisted only of the most conserved genes differentially abundant across all technical or analytical approaches, we overlapped the genes identified at week 2 in all three approaches, resulting in a 43-transcript signature. Functional analysis indicated over-representation of several innate immune signalling pathways, including TREM-1 (*P*=0.0002), TLR (*P*=0.0002), IL-1 (*P*=0.0009) and type I IFN (*P*=0.0022) (Fig. 5). A ‘weighted temporal molecular distance’ algorithm was further applied to quantify changes in abundance of these 43 transcripts over time (Fig. 5). The weighted temporal molecular distance algorithm measures the transcriptional perturbation of specified genes at a given time point relative to the mean at week 0. The non-IRIS patients had no significant changes in the level of the 43 genes at week 0, 0.5, 1 or 2. By contrast, there was significant transcriptional perturbation at week 2 in TB-IRIS patients compared with week 0 (*P*=0.0268). Furthermore, there was a significant difference in the transcript levels of these 43 transcripts between the TB-IRIS and the non-IRIS patients as early as 2–5 days post ART (week 0.5; *P*=0.008), before IRIS-associated clinical symptoms were apparent, suggesting that these genes are specifically associated with, and represent the earliest pathways involved in triggering clinical progression to, TB-IRIS.

### Inhibition of MyD88 reduces proinflammatory cytokines

To confirm the involvement of TLRs in the induction of cytokines that associate with TB-IRIS, we performed inhibition of the TLR signalling pathway. We chose to inhibit MyD88 in peripheral blood mononuclear cells (PBMCs) from both TB-IRIS and non-IRIS patients using a specific peptide inhibitor (Pepinh-MYD), compared with using a control peptide and RPMI medium control (mock).

**Figure 6:**
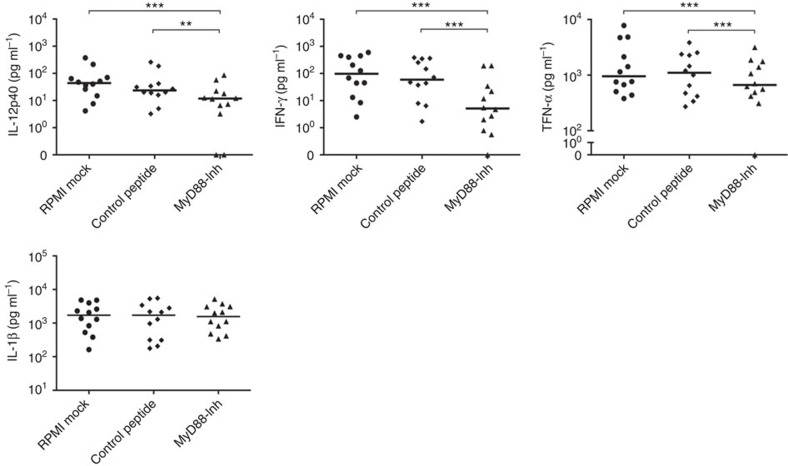
Inhibition of the MyD88 adaptor results in reduced cytokine productions. PBMC from TB-IRIS patients (week 2) were treated with MyD88 inhibitor (MYD88-Inh), control peptide or RPMI medium (mock), followed by stimulation with heat-inactivated H37Rv and the supernatant cytokine concentration was determined. An unstimulated background control was included for each treatment per sample. Data are shown as background subtracted (stimulated minus unstimulated) with a value of zero assigned to those negative after background subtraction. The Wilcoxon signed rank test was used for statistical comparisons. The median value of each group was shown and *P*-value was designated: *P≤0.05, **P≤0.01 and ***P≤0.001.

Among the analytes measured, concentrations of IL-12p40, IFN-γ and TNF-α in PBMC culture supernatant from TB-IRIS patients significantly decreased following treatment with MyD88 inhibitor, but not with the control peptide, compared with the RPMI mock (Fig. 6). The same experiments were also performed in the PBMCs from non-IRIS patients, but analytes were below the limits of detection. Similar observations have also been previously reported in the PBMCs from non-IRIS patients^28^. Although IL-1 was predicted to be activated downstream of the TLR-MyD88 signalling cascade, we failed to detect any difference in the level of IL-1β following MyD88 inhibition (Fig. 6), suggesting other immunological pathways are also involved in mediating the inflammatory response in TB-IRIS.

### Inflammasomes mediate proinflammatory responses in TB-IRIS

**Figure 7:**
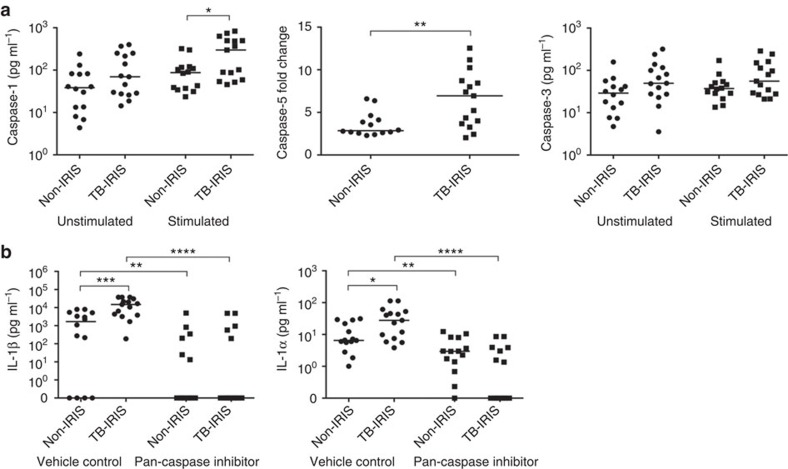
Both canonical and non-canonical inflammasomes are activated in TB-IRIS. (**a**) Patient PBMC from TB-IRIS and non-IRIS (week 2) were treated with pan-group 1 caspase inhibitor or dimethyl sulfoxide (DMSO) vehicle control, followed by stimulation with heat-inactivated H37Rv. An unstimulated background control was included for each treatment per sample. Concentrations of caspase-1 (left, on TC supernatant) and caspase-3 (right, with lysate) are shown as background subtracted (stimulated minus unstimulated) with a value of zero assigned to those negative after background subtraction. Caspase-5 (middle, with lysate) is expressed as fold-change of stimulated samples over unstimulated controls, as a standard curve was not available. The Wilcoxon signed rank test was used for statistical comparisons. The median value of each group was shown and *P*-value was designated: *P≤0.05, **P≤0.01, ***P≤0.001 and ****P≤0.0001. (**b**) Reflecting increased concentrations of caspase-1 and caspase-5, concentrations of IL-1β (left) and IL-1α (right) produced by patient PBMCs treated above were measured. Concentrations are expressed as that from MTB-stimulated minus the corresponding unstimulated control.

We next investigated TREM1 signalling, the other consistently over-represented pathway in TB-IRIS. The TREM1 activation in TB-IRIS consisted of upregulated messenger RNA expression of TLRs, NLR, NOD, CARD and CASP5 (caspase-5), all of which are involved in canonical and non-canonical inflammasomes. We detected a significantly higher concentration of caspase-1 in the supernatant of MTB-stimulated PBMC cultures from TB-IRIS patients at week 2 (Fig. 7a), suggesting the canonical inflammasome may be involved in mediating TB-IRIS. For the non-canonical inflammasome, PBMC lysates from TB-IRIS were found to have a significantly higher content of caspase-5, compared with non-IRIS (Fig. 7a), indicating that the non-canonical inflammasome is also activated during TB-IRIS. There was no significant difference in the anti-inflammatory apoptotic caspase-3 between TB-IRIS and non-IRIS (Fig. 7a). We next quantified the concentration of IL-1β and IL-1α in tissue culture (TC) supernatant as they are cleaved by caspase-1 and caspase-5, respectively. Significantly higher concentrations of IL-1β and IL-1α were detected in TB-IRIS, compared with non-IRIS (Fig. 7b). Treatment with an irreversible pan-caspase-1/4/5 inhibitor Z-WEHD-FMK significantly reduced the production of IL-1β and IL-1α in both groups, with a greater reduction observed in TB-IRIS. Together, these data suggest that inflammasome activation also contributes to the inflammatory response in TB-IRIS.

## Discussion

We have identified differentially abundant transcripts that characterize patients who develop TB-IRIS from those that do not as early as week 0.5 (days 2–5) following ART initiation, indicating an early transcriptional response before clinical symptoms. These early changes were predominantly related to enriched JAK/STAT signalling pathways mediated by either IL-6 or type I and II IFNs, as well as activation of PRRs and a role of macrophages via IL-1/18R, indicating the restoration of an innate immune response immediately following ART initiation. Although IL-1, IL-6, TNFα and IFN-γ have all been shown to be important in protection against MTB^29^,^30^,^31^,^32^,^33^, type I IFN may adversely affect the host immune response^34^. The transcriptomic signature of TB patients is characterized by a neutrophil-driven type I IFN-inducible response, which correlates with lung radiographic extent of disease and is downregulated on successful treatment^25^. Furthermore, in chronic MTB or *Mycobacterium leprae* infection, type I IFN plays an immunosuppressive role by inducing IL-10 and PDL1, and suppressing the production of IL-1α and IL-1β (refs 30, 35, 36, 37, 38). This inflammatory equilibrium of type I IFN appears to be disrupted in TB-IRIS, in which the negative feedback was not sufficient to control the downstream activation of the proinflammatory response, as we observed increased plasma concentration of IL-12p40 and enhanced secretion of IL-1α and IL-1β in PBMC cultures from TB-IRIS patients.

Of relevance, we also found that early differentially abundant transcripts in TB-IRIS predicted activation of nuclear factor-κB and p38-MAPK signalling pathways, and the production of proinflammatory cytokines and chemokines. The molecules of the week 0.5 signatures were also found to be upstream of the week 2 signatures. Indeed, we detected elevated plasma concentrations of some of these proinflammatory cytokines in TB-IRIS patients at week 2, compared with non-IRIS controls. These results are in agreement with our previous studies on TB-IRIS^18^,^28^,^39^,^40^.

We hypothesize that TB-IRIS arises from a combination of a high MTB antigen load at ART initiation^17^ and differential antigen recognition and immune signalling by innate immune receptors after ART initiation in TB-IRIS patients, which in turn contributes to hypercytokinemia and inflammation. Both TLR and IL-1R (Toll/IL-1 receptor (TIR)) share a 200-residue intracellular domain and activate a Rel-type transcription factor on stimulation^41^. In synergy with TIR, TREM1 modulates the production of cytokines and chemokines, and amplifies the proinflammatory response induced by TIRs^42^. Following bacterial sensing, TREM1 signals to activate downstream inflammasome activity^43^. MTB has been shown to activate caspase-1-dependent NLRP3 canonical inflammasome during infection that contributes to damaging innate inflammatory responses^44^,^45^. More recently, a non-canonical inflammasome has been described in Gram-negative bacterial infection that activates caspase-11 in mice (orthologue of caspase-4/5 in humans) through a lipopolysaccharide-dependent pathway^46^,^47^. Although MTB does not contain lipopolysaccharide, the bacilli have lipid-rich cell walls that contain complex glycosylated lipids, mycolic acids and acylated lipids, among others, and are readily sensed by TLR4 in response to infection^48^,^49^. We speculate that MTB may feasibly modulate the activity of non-canonical inflammasomes, as significantly higher levels of caspase-1 and caspase-5, and their prototypic cytokines IL-1β and IL-1α, were detected in PBMCs from TB-IRIS patients stimulated with heat-inactivated MTB, compared with the non-IRIS counterparts. A pan-group 1 caspase inhibitor, Z-WEHD-FMK, significantly reduced production of IL-1α and IL-1β, with a particularly prominent effect observed in TB-IRIS (Fig. 7), further indicating that the proinflammatory response in TB-IRIS is at least in part mediated by inflammasomes. It is established that TLRs and inflammasome signals converge to amplify the innate immune response^50^. More recently, this convergence in signalling has also been shown to amplify CD4^+^ T-cell effector functions in bacterial pathogen clearance in a MyD88-dependent manner^51^. With the exception of TLR3, all other TLRs and IL-1R signal through the MyD88 adaptor. Inhibition of the MyD88 in PBMCs from TB-IRIS patients significantly reduced the production of proinflammatory cytokines (Fig. 6), indicating that a TLR-MyD88 signalling pathway contributes to the dysregulated cytokine response in TB-IRIS. Interestingly, PBMCs from the non-IRIS patients produced minimal levels of IFN-α, IFN-γ, IP-10 and IL-12, following stimulation, and inhibition of the MyD88 adaptor did not reduce production of other cytokines/chemokines, further suggesting that the dysregulated cytokine/chemokine production via MyD88 signalling contributes to TB-IRIS. Together, our data provide good evidence that inflammation and subsequent T-cell expansion in TB-IRIS is driven by innate immune signalling and its amplification, which triggers the activation of transcriptional factors, with the end result being hypercytokinemia contributing to systemic inflammation.

Two recent studies on TB-IRIS also investigated the role of innate immunity via microarray analysis of blood monocytes^24^,^52^. A few of the differentially expressed genes in TB-IRIS from these studies were also identified in ours, which used whole blood. The authors of these studies also reported PRRs, the complement system and communication between innate and adaptive immunity as among the most significantly upregulated pathways, further suggesting a prominent contribution of innate immunity to the pathogenesis of TB-IRIS.

Prednisone is used for the treatment of TB-IRIS and has been demonstrated in a clinical trial, to result in more rapid clinical improvement^53^. However, corticosteroids have potential side effects in patients with advanced HIV-related immunosuppression such as the development of herpes zoster and Kaposi’s sarcoma^54^,^55^, and therefore therapeutic strategies that offer greater immune specificity should be explored. We acknowledge that our sample size is small and our findings require further validation; nevertheless, the ability to identify a transcriptomic signature of TB-IRIS within days of commencing ART may be beneficial in directing preventive interventions in the absence of a pre-ART predictive biomarker. We have demonstrated that blocking MyD88 and group 1 caspases reduced the production of proinflammatory cytokines in TB-IRIS. Based on our findings, we postulate that immunomodulators have the potential to interrupt the inflammatory pathways that lead to TB-IRIS and their utility as part of the prevention strategies of TB-IRIS could be explored in future studies. Anti-inflammatory agents that suppress innate signalling networks are currently being evaluated in clinical trials as treatment options for autoimmune diseases^56^. An anti-IL-1β monoclonal antibody (canakinumab) is approved for use in autoinflammatory syndromes and recombinant IL-1R antagonist (anakinra) for rheumatoid arthritis, suggesting a potential role for these drugs in other inflammatory conditions. HIV-TB coinfected patients who display an early transcriptomic signature associated with an increased risk of developing TB-IRIS may potentially benefit most from such pre-emptive therapies. In this respect, the recent description of MCC950 (ref. 57), a potent, selective, small-molecule inhibitor of NLRP3, is of particular interest.

## Methods

### Ethical approval

The University of Cape Town’s Faculty of Health Sciences Research Ethics Committee approved this study (HREC No: 049/2009). All participants provided written informed consent.

### Study design and participant recruitment

Hospitalized patients with HIV-associated TB were recruited to a prospective, observational cohort study at Brooklyn Chest Hospital (Cape Town, South Africa) between April 2009 and December 2010 (refs 39, 58). Eligible patients were adults older than 18 years of age with serologically confirmed HIV-1 infection and a diagnosis of PTB and/or EPTB. Patients were ART naive at the time of recruitment and started ART according to WHO guidelines (CD4 count <200 cells per mm^3^ or WHO stage 4 defining illness before March 2010 and CD4 count <350 cells per mm^3^ or WHO stage 4 defining illness thereafter). Exclusion criteria included patients with known rifampicin-resistant TB and inability to provide consent. We selected the first 32 patients (17 TB-IRIS and 15 non-IRIS) enroled, from whom longitudinal Tempus blood samples were available to undertake microarray analysis. We further included another 31 patients (16 TB-IRIS and 15 non-IRIS) to analyse plasma proteins and investigate potential mechanisms mediating TB-IRIS. All but one patient, who was prescribed nevirapine, received efavirenz-based non-nucleoside reverse-transcriptase inhibitor containing ART regimens. Ten patients (four TB-IRIS and six non-IRIS) were on corticosteroid therapy when they commenced ART for an average of 41 days. Corticosteroids were prescribed to most participants who were diagnosed with TB-IRIS.

### Case definitions

TB was diagnosed on the basis of smear or culture positivity. Where this was negative or unavailable, diagnosis was according to WHO guidelines for diagnosis of smear negative or EPTB in HIV-1-infected persons^59^. TB-IRIS was diagnosed according to the International Network for the Study of HIV-associated IRIS case definition^60^. TB-IRIS patients typically displayed systemic and/or local inflammatory features including fever, weight loss, tachycardia, lymphadenitis, enlarging serous effusions and new or recurrent infiltrates on chest radiographs^60^. Non-IRIS patients were defined as those free of IRIS symptoms during the 3-month follow-up period.

### Blood sample collection and processing

Whole blood was collected in sodium-heparin and Tempus tubes (Life Technologies, Carlsbad, CA) longitudinally at four different timepoints: week 0 before initiation of ART and weeks 0.5 (2–5 days post-ART), 1 and 2 for both TB-IRIS and non-IRIS patients post ART. Except for patients who were already receiving corticosteroids at the time of ART initiation, all other patient blood samples before and at week 2 were taken before initiation of corticosteroid therapy. Plasma and PBMCs were separated from whole blood collected in sodium-heparin using Ficoll-Paque (Pharmacia Biotech, Uppsala, Sweden) according to the manufacturer’s protocol. Plasma was stored at −80 °C and PBMCs were stored in the vapour phase of liquid nitrogen until use. Whole-blood and plasma samples were not available for all time points in some patients because of their discharge or transfer.

### RNA extraction and processing for microarray analysis

RNA was extracted from whole blood in Tempus tubes using the MagMAX for Stabilized Blood Tubes RNA Isolation kit (Life Technologies) according to the manufacturer’s instructions. Total RNA (2.5 μg) was then globin reduced using the GLOBINclear 96-well plate format kit (Life Technologies). The total RNA yield after globin reduction was determined in a NanoDrop 1,000 spectrophotometer (Fisher Scientific, Waltham, MA) and integrity assessed by Agilent 2,100 Bioanalyzer (Agilent Technologies, Santa Clara, CA). Samples with RNA integrity number >6.0 were determined to be of acceptable quality for further analysis. Approximately 250 ng of globin-reduced RNA was used to prepare amplified and biotinylated antisense complementary RNA targets using the Illumina TotalPrep RNA amplification kit (Life Technologies). Seven hundred and fifty nanograms of labelled cRNA was hybridized overnight to Illumina human HT-12 v4 BeadChip arrays (Illumina Inc., San Diego, CA), which contained >47,000 probes (transcripts). Some genes were represented by more than one probe (transcript). The arrays were then washed, blocked, stained and scanned on an Illumina BeadStation 500. Signal intensity values from the scans were generated by the Illumina BeadStudio v2 software. All samples were randomized during all stages of processing to avoid any batch effect.

### Microarray data analysis

A detail flow diagram on the analytical strategy is shown in Supplementary Fig. 1. Raw microarray data were background subtracted and scaled data were generated using Illumina BeadStudio v2 software. A stringent filter was implemented to select only those transcripts present in 100% of all samples in either the IRIS or the non-IRIS group. The transcripts were further selected to have ≥2-fold expression change compared with the non-IRIS samples. The Mann–Whitney *U*-test was applied and corrected with asymptotic Benjamini–Hochberg false discovery rate set at *P*=0.05. The identified transcripts were then clustered using a hierarchical algorithm with Pearson’s uncentred distance metric and average linkage.

Canonical pathway, upstream regulator and molecule activity prediction of differentially abundant transcripts were analysed by Ingenuity Pathway Analysis (Ingenuity Systems, www.ingenuity.com). The significance (*P*-value) of association between the transcripts and the pathway was calculated using Fisher’s exact test of probability. The weighted temporal molecular distance was determined for each time point as previously described^61^, except that the mean baseline value of each group was used as a control. Briefly, the algorithm determined whether the raw expression value of each differentially expressed transcript in each sample lay within or outside two s.d. of the baseline mean. To qualify, transcripts had to differ by at least 200 units in raw signal intensity and 2 s.d. from the baseline mean. The weighted distance is the group mean of the sum of the total s.d. for all qualifying transcripts in each sample in each patient.

### NanoString

Differentially abundant transcripts identified by microarray analysis were reinvestigated and validated using a customized NanoString nCounter assay (NanoString Technologies, Seattle, WA)^62^. Housekeeping genes (*n*=9) and customized barcoded capture/reporter probe pairs specific for each transcript (*n*=124) were hybridized overnight at 65 °C to 100 ng of total RNA samples previously used in microarray. Six additional positive and negative control probe pairs were also included. Unhybridized probes were removed and the hybridized probes were purified on an nCounter Prep Station. The barcode on each reporter probe was scanned with an nCounter Digital Analyzer to generate a quantitative measure of the hybridized RNA. Sample signal values were background subtracted and normalized first to the positive control spikes and then to housekeeping genes. Ninety-nine of the 107 samples from week 0, 0.5, 1 and 2 were included when sufficient RNA was available.

### Plasma protein determination

The plasma concentrations of 18 cytokines and chemokines were measured in 33 TB-IRIS and 30 non-IRIS patients in week 0 and week 2 samples. TNF-α, IFN-γ, IFN-α2 and IL-1β, IL-6, IL-8 and IL-12p40 were quantified on the Bio-Plex platform (Bio-Rad Laboratories, Hercules, CA), using customized Milliplex kits (HCYTMAG-60 K, Merck Millipore, Germany). The concentration of IL-1α was measured by enzyme-linked immunorsorbent assay (eBioscience, San Diego, CA).

### MyD88 inhibition assay

PBMCs from 13 TB-IRIS and non-IRIS patients (week 2, microarrayed) were defrosted and rested overnight at 37 °C in RPMI with 10% FCS. Total cell number was counted using a Coulter Counter (Beckman, Brea, CA) and cell viability was determined by Trypan blue staining in a TC-20 automatic cell counter (Bio-Rad, Hercules, CA). Lyophilized MyD88 peptide inhibitor (Pepinh-MYD; InvivoGen, San Diego, CA) and a control peptide (Pepinh-Control; InvivoGen) were dissolved in endotoxin-free water at 1 mM stock concentration. PBMCs were treated with MyD88 peptide inhibitor (50 μM), control peptide (50 μM) or RPMI mock for 5 h at 37 °C. Samples were either stimulated or unstimulated with heat-inactivated MTB (H37Rv; multiplicity of infection 1:1) overnight at 37 °C. TC supernatant was harvested and purified by centrifugation twice at 2,000 r.p.m. and stored at −80 °C until use. Cytokine concentrations in supernatants were quantified by Luminex analysis. Heat-inactivated H37Rv was used, because patients had undergone intensive antibiotic therapy at the time of sampling and any antigens present would probably be bacterial fragments rather than actively replicating bacilli.

### Group 1 caspase inhibition assay

PBMCs from 16 TB-IRIS and 15 non-IRIS patients (week 2, non-microarrayed) were thawed, rested and counted as above. Lyophilized caspase-1/4/5 irreversible peptide inhibitor (Z-WEHD-FMK; R&D Systems, Minneapolis, MN) was dissolved in dimethyl sulfoxide at 1 mM stock concentration. PBMCs were treated with the Z-WEHD-FMK peptide inhibitor (50 μM) or dimethyl sulfoxide vehicle control (50 μM) for 1 h at 37 °C. Supernatant was then removed and replenished with a fresh stock of RPMI supplemented with 10% FCS. Samples were either stimulated or unstimulated with heat-inactivated MTB (H37Rv; multiplicity of infection 1:1) overnight at 37 °C. TC supernatant was harvested and purified by centrifugations twice at 2,000 r.p.m. The residual PBMC pellets were lysed in lysis buffer on ice for 1 h and recentrifuged to remove all cell debris. Cytokine concentrations in supernatants were quantified by Luminex assay. Concentrations of caspase-1 (R&D Systems) and IL-1α (eBioscience) in supernatant and caspase-3 (eBioscience) and caspase-5 (BioVision, Milpitas, CA) in PBMC lysate were measured by enzyme-linked immunorsorbent assay. The activity of caspase-5 was expressed as the fold change in absorbance (OD_405nm_) in MTB-stimulated over unstimulated PBMC lysate, as recombinant protein standards were not supplied in the assay kit.

### Statistical analysis

Statistical analyses were performed in GraphPad Prism 6.0 software. Patient baseline characteristics were compared by *χ*^2^-test for contingency data or Kruskal–Wallis test for continuous data. The weighted temporal molecular distance between IRIS and non-IRIS patients was compared using the unpaired *t*-test with Welch’s correction. Differences in cytokine concentrations between patient or treatment groups were compared by Mann–Whitney *U*- test or Wilcoxon signed rank test for paired data. Potential significance was inferred for results associated with a *P*-value of ≤0.05. Microarray data and canonical pathway analysis were corrected for multiple testing using Benjamini–Hochberg false discovery rate with *q*=0.05.

## Additional information

**Accession codes:** The microarray data has been deposited in the NCBI’s Gene Expression Omnibus under GEO Series accession number GSE58411.

**How to cite this article:** Lai, R. P. J. *et al.* HIV–tuberculosis-associated immune reconstitution inflammatory syndrome is characterized by Toll-like receptor and inflammasome signalling. *Nat. Commun.* 6:8451 doi: 10.1038/ncomms9451 (2015).

## Supplementary Material

Supplementary Information Supplementary Figure 1 and Supplementary Tables 1-6
