# Supplementary material for: HIV–tuberculosis-associated immune reconstitution inflammatory syndrome is characterized by Toll-like receptor and inflammasome signalling
Source: Nat Commun. 2015 Sep 24;6:8451. doi: 10.1038/ncomms9451 (PMC4595995; doi:10.1038/ncomms9451)
Supplement: Supplementary Information — Supplementary Figure 1 and Supplementary Tables 1-6 [file ncomms9451-s1.pdf]

Supplementary Figure 1. Microarray data mining and validation

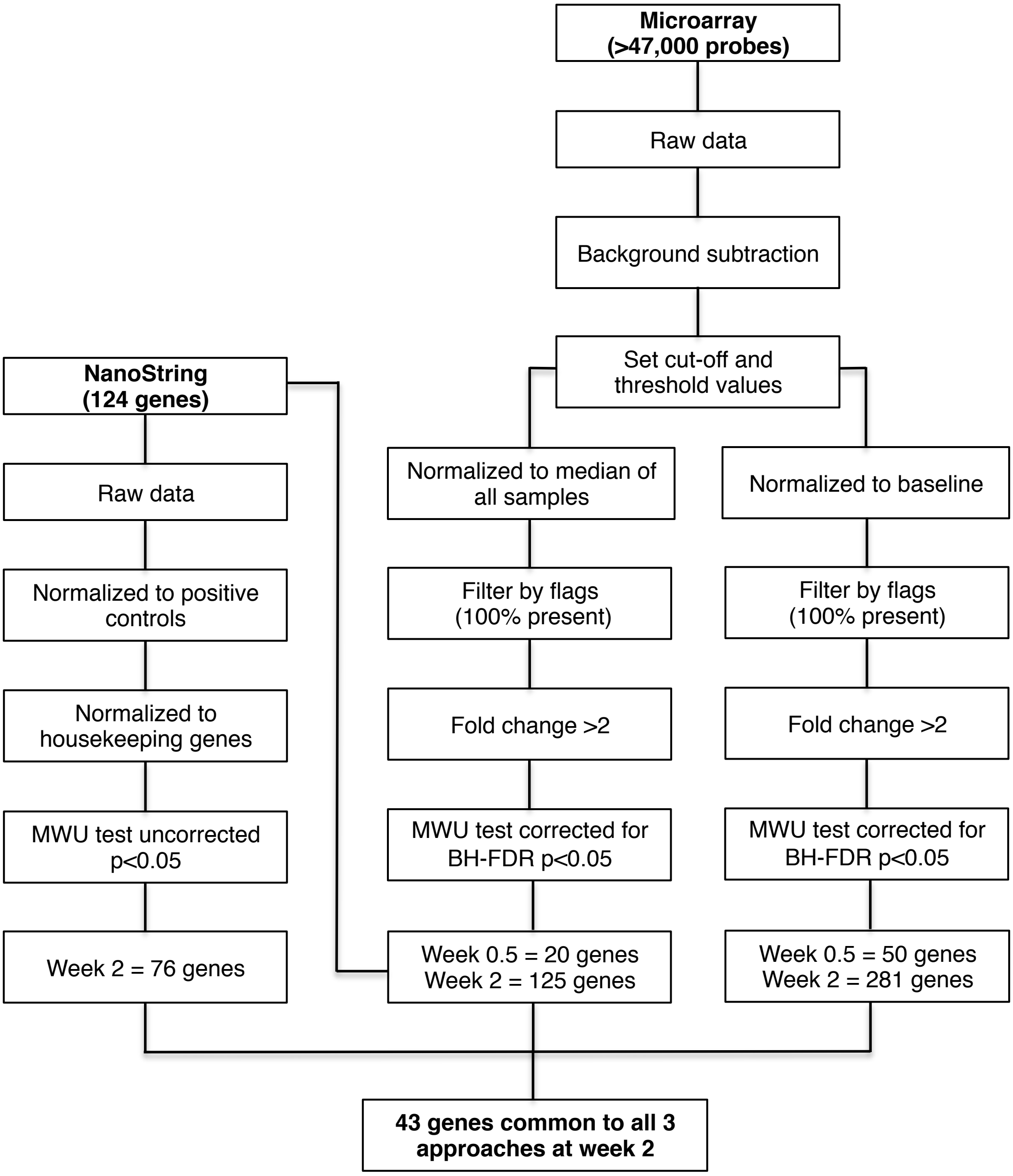

Two analytical approaches were used to extract differentially abundant transcripts from microarray. The classical approach is by normalization to median of all samples and by our alternative approach, normalization to individual baseline values. Genes from the classical approach were then validated by a technically independent method, the NanoString assay. At 2 weeks, 43 transcripts were consistently differentially abundant irrespective of platform and analytic method. MWU = Mann Whitney U test; BH-FDR = Benjamini-Hochberg corrected False Discovery Rate.

Supplementary Table 1. Clinical characteristics of 63 patients with HIV-associated TB

Variables	Microarray		Non-microarray		*P*-value
	**Non-IRIS (n = 15)**	**IRIS (n = 17)**	**Non-IRIS (n = 15)**	**IRIS (n = 16)**	
Male sex	6 (35)	6 (40)	7 (44)	7 (47)	0.923
Age (years)	28.8 (27-37)	31 (27-36)	32.6 (24-43)	32.9 (26-44)	0.770
TB treatment prior to ART (days)	41 (34-76)	38 (25-64.5)	26 (22-43)	54 (27-62)	0.098
HIV viral load (10^4 copies/ml)	26 (6.1-72)	32 (13-80.5)	49 (27-250)	102 (15-158)	0.285
CD4 baseline (cells/mm^3)	96 (27-156)	80 (35-151)	82 (50-205)	88 (40.5-112)	0.907
**TB Diagnosis**					0.738
Clinical & Radiological	5 (33)	4 (24)	4 (27)	5 (31)	
Sputum smear	0 (0)	0 (0)	0 (0)	1 (6)	
Cultured AFB/MTB	10 (67)	13 (76)	11 (73)	10 (63)	
**Type of TB**					0.605
PTB	2 (13)	3 (18)	2 (13)	2 (12)	
EPTB	5 (33)	1 (6)	2 (13)	3 (19)	
PTB + EPTB	8 (63)	13 (76)	11 (73)	11 (69)	
**WHO stage**					0.946
III	2 (13)	2 (12)	2 (13)	3 (19)	
IV	13 (87)	15 (88)	13 (87)	13 (81)	
Pre-ART corticosteroid therapy	2 (13)	2 (12)	4 (27)	2 (13)	0.630

TB-IRIS and non-IRIS patients included in the analysis were found to be matched for clinical variables in subsequent analysis. Numbers in parentheses are percentage for contingency data or median plus interquartile range for continuous data.

Supplementary Table 2. Differentially abundant transcripts identified at week 0.5

Week 0.5 (Normalized to median)		
**Gene**	**Regulation**	**Fold change**
BATF2	up	2.1846
CEACAM3	up	2.0467
DHRS13	up	2.0908
FCGR1A	up	2.0983
FCRL2	down	2.0634
FUT6	up	2.5618
HIST1H2BD	up	2.1440
HIST1H3D	up	2.0573
HS.580797	up	2.0555
IER3	up	2.0587
LOC653737	up	2.9497
LOC728744	up	2.4095
LRG1	up	2.0708
MAFF	up	3.2146
OSM	up	2.1419
PID1	down	2.0222
SERPING1	up	2.5423
SOCS1	up	2.4922
SOCS3	up	2.3873
TIMM10	up	2.1626

Week 0.5 (Normalized to baseline)		
**Gene**	**Regulation**	**Fold change**
AIM2	up	2.0037
ANP32A	up	2.1600
APOL2	up	2.3902
APOL6	up	2.1582
BATF2	up	2.4047
BCL2A1	up	2.5283
BST2	up	2.0929
CARD16	up	2.3954
CEACAM1	up	2.1052
CEACAM3	up	2.1852
DDAH2	up	2.0794
DDX60L	up	2.0863
DHRS9	up	2.4663
FCGR3A	up	2.0743
FCHO2	up	3.4417
GBP3	up	2.2201
GRAMD1B	up	2.1225
HIST1H3D	up	2.5532
HIST1H4E	up	2.2033
HIST1H4H	up	2.0072
HLX	up	2.0780
HSPA1B	up	2.2859
IFI35	up	2.1308
IFIH1	up	2.0581
IFIT2	up	2.2617
IFIT3	up	2.7773
IL18R1	up	2.3164
IL1RN	up	2.0761
IRF7	up	2.5627
LOC100128274	up	2.0506
LOC100129681	up	2.3055
MDK	up	2.0850
NCF1B	up	2.0535
NCOA1	up	2.3165
NT5C3	up	2.0320
OAS1	up	3.8800
OASL	up	2.8671
PLSCR1	up	2.3430
RAB33B	up	2.0973
RSAD2	up	2.7584
RTP4	up	2.3444
SAMD9L	up	2.2535
SEPT4	up	2.2749
SERPING1	up	2.5142
SIPA1L2	up	2.6676
SOCS1	up	2.5847
SP140	up	2.2170
TNFSF13B	up	2.2225
USP25	up	2.0829
ZBP1	up	2.0378

Using two different normalization approaches, we identified 20 (normalized to median) and 50 (normalized to baseline) genes that were differentially abundant in TB-IRIS. Five genes overlapped in both approaches: BATF2, CEACAM3, HIST1H3D, SERPING1 and SOCS1.

Supplementary Table 3. Differentially abundant transcripts identified at week 2 (normalization to median)

Week 2 (Normalized to median)					
Gene	Regulation	Fold change	Gene	Regulation	Fold change
ACSL1	up	2.1155	LILRA5	up	2.1281
ADCY3	up	2.3588	LIMK2	up	2.2041
ADM	up	2.0180	LIN7A	up	2.1097
AGPAT9	up	2.1554	LOC100128460	up	3.3478
AIG1	up	2.1108	LOC100130562	up	2.0700
ANXA3	up	2.5022	LOC100132499	up	2.3290
APOB48R	up	3.3717	LOC100133177	up	2.6813
AQP10	up	2.1727	LOC100170939	up	2.0604
ASPRV1	up	2.0847	LOC642103	up	2.2657
B3GNT8	up	3.1768	LOC642469	down	3.9874
BAMBI	up	2.4099	LOC642684	up	2.1258
BASP1	up	2.0408	LOC644852	down	3.3307
BCL2A1	up	2.0503	LOC648984	up	2.1752
C19orf35	up	2.3152	LOC653604	up	2.2750
C19orf59	up	4.6613	LOC728519	up	2.0439
CAMKK2	up	2.0367	LRG1	up	2.1396
CARD17	up	2.5862	LRPAP1	up	2.4931
CASP5	up	2.8438	MAFF	up	2.8657
CATSPER1	up	3.0975	MANSC1	up	2.2427
CDK5RAP2	up	2.8255	MAPK13	up	2.0964
CEACAM1	up	2.3311	MAPK14	up	2.7632
CEACAM3	up	2.0294	MIAT	down	2.0175
CKAP4	up	2.1239	MIIP	up	2.9883
CLC	down	2.1189	MMP25	up	2.0077
COL4A3BP	up	2.0594	MRPL40	up	2.0012
CR1	up	2.0422	NAMPT	up	2.0470
CSF3R	up	2.1805	NBN	up	2.2320
CYP1B1	up	2.9635	NLRC4	up	2.0893
DHRS13	up	2.1028	OAS1	up	2.4606
DKFZp761E198	up	2.1435	PAG1	up	2.0545
DOK3	up	2.0834	PFKFB3	up	2.0463
DSC2	up	2.0717	PGS1	up	2.0428
ECE1	up	3.1570	PHAX	up	2.9282
EEF1D	up	2.1216	PPP2R3A	up	3.3511
ELF2	up	2.3019	PRDX6	up	2.1671
ERI1	up	2.1700	PSG9	up	2.4055
FCGR1A	up	2.0686	RAB20	up	2.1387
FCGR1C	up	2.0742	RASGRP4	up	2.1070
FCRL2	down	2.0786	RBM47	up	2.0524
FLJ20273	up	2.1516	RHOBTB1	up	2.0792
FLJ35801	down	2.0398	RP2	up	2.1630
FLJ43093	down	2.0929	SEMA6B	down	2.0371
FUT6	up	2.3314	SERPINA1	up	2.5439
GK	up	2.2046	SERPING1	up	2.5050
GLT1D1	up	2.4152	SIGLEC9	up	2.4710
GNB4	up	2.6021	SIPA1L2	up	2.9022
GPR141	up	2.0701	SMARCD3	up	2.8686
GPR160	up	2.1084	SPRYD3	down	2.5933
GPR175	down	2.3622	SPTY2D1	up	2.0371
GPR97	up	2.4049	TDRD9	up	2.6342
H2AFJ	up	2.0541	TLR10	up	2.4742
HECW2	up	2.3920	TLR2	up	2.6256
HIST1H2BD	up	2.3411	TLR4	up	2.0213
HIST1H4H	up	2.0331	TLR5	up	2.3194
HIST2H2AB	up	3.1505	TP53I3	up	2.0646
HIST2H2AC	up	2.4071	TPST1	up	4.4383
IFIT3	up	2.0150	TRIB1	up	2.0703
IFITM3	up	2.1029	TUFT1	up	2.5390
IGSF6	up	2.1114	VNN1	up	3.0706
IL18R1	up	2.4438	ZAK	up	2.5763
IL1R2	up	2.8923	ZBTB16	up	2.1318
KCNJ15	up	2.0738			
KIAA0232	up	2.2386			
KLHL2	up	2.1108			

Using the normalization to median approach, 125 genes were identified to be differentially abundant in TB-IRIS at week 2 (median time of symptom onset).

Supplementary Table 4. Differentially abundant transcripts identified at week 2 (normalization to baseline)

Week 2 (Normalized to baseline)											
Gene	Regulation	Fold change	Gene	Regulation	Fold change	Gene	Regulation	Fold change	Gene	Regulation	Fold change
ACSL1	up	2.3256	FCHO2	up	2.4366	LOC100129685	down	2.5298	RAB33B	up	2.1629
ADCY3	up	2.3680	FCRL2	down	2.0023	LOC100129960	up	2.0815	RANBP10	down	2.1423
ADORA1	down	2.2684	FKBP4	up	2.6435	LOC100130905	down	2.2309	REPS2	up	2.1569
AGPAT9	up	2.2578	FKBP5	up	2.0925	LOC100131205	down	2.0947	RHOBTB1	up	2.2040
AIG1	up	2.1336	FLJ20273	up	2.2869	LOC100133875	up	2.2971	RNF187	down	2.0793
AIM2	up	2.2608	FLJ22662	up	2.0312	LOC153561	down	2.0189	RP11-529I10.4	down	2.5855
AK1	down	2.1047	FLJ43093	down	2.7362	LOC201175	up	2.0344	RPL12P6	down	2.1363
ALAS2	down	2.1613	FOXO3	down	2.5466	LOC400174	down	2.1134	RSAD2	up	2.0511
ALDOB	down	2.0971	FTHL2	up	2.2779	LOC440731	up	2.0481	RTP4	up	2.1332
ALPL	up	3.0872	FYB	up	2.6117	LOC441455	down	2.5082	S100A11	up	2.0180
ANP32A	up	2.5415	GBP3	up	2.5565	LOC641922	up	2.0912	S100A12	up	2.6416
ANXA3	up	2.5425	GFOD2	down	2.0079	LOC641972	down	2.2380	S100P	up	3.2420
APOB48R	up	3.5853	GNB4	up	2.6133	LOC642684	up	2.4548	SAMD9L	up	2.1760
ARHGAP18	up	2.2319	GPR141	up	2.3506	LOC646674	down	2.0767	SAMSN1	up	2.2845
ASPRV1	up	2.2132	GPR160	up	2.2312	LOC646786	up	2.2434	SDF2	up	2.0732
AVL9	up	2.3318	GPR97	up	2.3020	LOC648984	up	2.0573	SELS	up	2.0285
AXUD1	up	2.1984	GYG1	up	2.3394	LOC652968	down	2.0642	SERPINB8	up	2.4954
B3GNT8	up	3.2140	H2AFJ	up	2.2217	LOC653156	down	2.2411	SERPING1	up	2.8118
B4GALT5	up	2.0341	HDC	down	2.7073	LOC653604	up	2.1250	SIGLEC7	up	2.2769
BAMBI	up	2.3700	HECW2	up	3.1726	LOC653610	up	2.5061	SIGLEC9	up	2.0724
BASP1	up	2.0922	HERC5	up	2.2221	LOC730278	up	2.1955	SIPA1L2	up	3.5709
BATF	up	2.0174	HIST1H1C	up	2.0388	LRIG1	down	2.0420	SLC11A1	up	2.3418
BCL11B	down	2.2484	HIST1H2BD	up	2.5245	LRPAP1	up	2.5161	SLC14A1	down	2.2494
BCL2A1	up	2.7513	HIST1H3D	up	2.3443	LY6E	up	2.3120	SLC1A5	down	2.0889
BCL6	up	2.1054	HIST1H4E	up	2.9186	LY96	up	2.1666	SLC25A45	down	2.2426
BSG	down	2.2896	HIST1H4H	up	2.7948	MAL	down	2.7359	SLC26A8	up	2.2333
BST2	up	2.1840	HIST1H4K	up	2.1948	MANSC1	up	2.1736	SMARCD3	up	2.6609
C10orf119	up	2.8379	HIST2H2AA3	up	2.3654	MAP2K3	down	2.1504	SOAT1	up	2.2423
C14orf45	down	2.1259	HIST2H2AA4	up	2.2571	MAPK14	up	3.4301	SP100	up	2.1176
C19orf35	up	2.5482	HIST2H2AB	up	4.0097	MARCH8	down	2.6375	SPAST	up	2.0536
C19orf59	up	5.1205	HIST2H2AC	up	2.8817	MAZ	up	2.4779	SPATS2L	up	2.1361
C1orf128	down	3.1957	HIST2H4A	up	2.1881	MFF	up	2.6520	SPTLC2	up	2.3358
CARD16	up	2.4014	HPS1	down	2.3230	MIA3	up	2.3351	SRRD	down	3.3025
CARD17	up	2.5771	HPSE	up	2.0011	MICALCL	down	2.0337	SSBP3	down	2.2848
CARS	up	2.1578	HSPA1B	up	2.0191	MIIP	up	2.8756	STRADB	down	2.0523
CASP5	up	2.1071	IDO2	down	2.1109	MIR877	down	2.1096	STX3	up	2.1020
CATSPER1	up	3.6649	IFI44	up	2.1644	MMP9	up	2.4137	SULT1B1	up	2.0547
CCNJL	up	2.8942	IFI44L	up	2.0602	MOSC1	up	2.3845	SYTL4	up	2.0718
CDK5RAP2	up	2.7798	IFI6	up	2.6068	MSI2	down	2.0635	TCP11L2	down	3.5195
CEACAM1	up	2.1721	IFIT1	up	2.1166	MX1	up	2.2036	TDRD9	up	3.2307
CLEC1B	up	2.4012	IFIT1L	down	2.3053	NACC2	up	2.2815	TESC	down	2.2966
CLTC	up	2.8029	IFIT2	up	2.1212	NBN	up	2.3769	TFDP2	down	2.0391
COL4A3BP	up	2.2222	IFIT3	up	2.7371	NCOA1	up	2.3029	TIFA	up	2.0736
CSTA	up	2.2791	IFITM3	up	2.1774	NFIA	down	2.2776	TIMM10	up	2.3443
CXCL1	up	2.1209	IL18R1	up	2.9501	NFIL3	up	2.0041	TLR2	up	2.4957
DCAF6	down	2.2202	IL1RN	up	2.1560	NFIX	down	2.0711	TLR5	up	2.2334
DDAH2	up	2.5038	IRAK3	up	2.2114	NINJ2	down	2.6326	TNFSF10	up	2.0966
DDX60L	up	2.0438	IRF7	up	2.9951	NSUN7	up	2.0561	TNS1	down	2.4877
DENND1A	up	2.3557	ISG15	up	3.4062	NT5C3	up	2.0171	TP53I3	up	2.4066
DHRS13	up	2.1181	JUN	up	2.1901	OAS1	up	3.3915	TPST1	up	7.5454
DHRS9	up	2.1519	KCNJ10	down	2.0959	OAS2	up	2.5120	TRAM2	up	2.1697
DHX58	up	2.3065	KCNJ15	up	2.3010	OAS3	up	2.3832	TREML2	up	2.0346
DKFZp761E198	up	2.1409	KIAA0319L	up	2.4675	OASL	up	2.8053	TRIB1	up	2.3092
DNM1L	up	2.5864	KIAA1033	up	2.3233	OMA1	up	2.6006	TRIM10	down	2.6405
DOCK5	up	2.0903	KIAA1618	up	2.4717	OPLAH	up	3.5658	TRIM25	up	2.1102
DOK3	up	2.2972	KIF1B	up	2.2957	OR2W3	down	3.1770	TSPAN5	down	2.3140
DPM2	down	2.4343	KLC3	down	2.9126	PACSIN2	up	2.0369	TSPAN7	down	2.4124
DYSF	up	2.0009	KLF1	down	2.5882	PCSK1N	down	3.4752	TTC25	down	2.9449
E2F2	down	2.1564	KLHL2	up	2.4385	PDZK1IP1	down	2.5859	TTC33	up	2.1756
ECE1	up	2.4754	KLHL8	up	2.1408	PFKFB3	up	2.3180	TTRAP	up	3.6305
EEF1D	up	2.1554	LAMC1	up	2.3499	PHC2	up	2.0296	TUFT1	up	2.8016
EIF2AK2	up	2.4413	LAMP3	up	2.9038	PHF3	up	2.2618	UBE2R2	up	2.2785
EPB41	down	2.0897	LEF1	down	2.3518	PIP5K2A	down	2.2079	UBL5	up	2.1610
EPB42	down	2.3718	LGALS9	up	2.2138	PLSCR1	up	2.7941	USP48	up	3.1787
EXOSC4	up	2.0938	LILRA3	up	2.3850	PMAIP1	up	2.0289	VNN1	up	3.3758
F5	up	2.0405	LILRA6	up	2.1326	PPP2R3A	up	2.8525	ZFP36L1	up	2.0076
FBXO30	up	2.5710	LIN7A	up	2.9365	PRDX2	down	2.4654	ZNF23	down	2.6074
FBXO7	down	2.2887	LMNB1	up	2.0160	PRDX6	up	2.9741	ZNF683	down	2.6651
FBXO9	down	2.4332	LOC100128460	up	2.7702	PSG9	up	3.0580			
FCER1A	down	2.9629	LOC100129674	up	2.2387	PSMF1	down	2.0079			
FCGR3A	up	2.1977	LOC100129681	up	2.4017	PTGDR	down	2.0095			

Using the normalization to baseline approach, 281 genes were identified to be differentially abundant in TB-IRIS at week 2 (median time of symptom onset).

Supplementary Table 5. Upstream activity prediction of week 2 signature

Upstream Regulator	Molecule Type	Predicted Activation State	Activation z-score	p-value of overlap
IRF7	transcriptional regulator	Activated	2.747	1.32E-06
OSM	cytokine	Activated	3.379	3.08E-05
IFNG	cytokine	Activated	3.534	4.81E-05
IRF3	transcriptional regulator	Activated	2.176	6.49E-04
TNF	cytokine	Activated	3.051	7.85E-04
CSF3	cytokine	Activated	2.215	1.90E-03
HIP1A	transcriptional regulator	Activated	2.195	9.77E-03
IL-5	cytokine	Activated	2.213	1.37E-02
CEBPA	transcriptional regulator	Activated	2.235	1.78E-02
IL1B	cytokine	Activated	2.117	4.61E-02

Upstream regulator prediction was performed using the transcriptomic signatures identified at week 2 (normalization to median method). An array of proinflammatory cytokines and transcription factors that regulate interferon and other cytokines were predicted to be activated (see also Figure 3). The activation *z*-score was calculated by matching observed and predicted up/down-regulation patterns based on the signatures. The *p*-value of overlap measures overlap of observed and predicted regulated gene sets using the Fisher’s exact test.

Supplementary Table 6. Validation of microarray data by NanoString assay

Gene	Mean (non-IRIS)	Mean (TB-IRIS)	P value	Significance
ACSL1	5370.53	12095.20	0.0020	**
ADCY3	113.95	255.62	0.0385	*
ADM	354.33	823.36	0.0043	**
AGPAT9	59.29	97.62	0.0161	*
AIG1	107.60	168.82	0.0055	**
ANXA3	638.62	1513.45	0.0070	**
APOB48R	36.66	55.16	0.0240	*
AQP10	28.24	54.15	0.0167	*
ASPRV1	38.74	78.11	0.0007	***
B3GNT8	32.13	65.21	0.0011	**
BAMBI	19.05	39.55	0.0019	**
BASP1	2591.31	5819.50	0.0036	**
BCL2A1	726.43	1348.78	0.0114	*
C19orf35	52.11	95.88	0.0095	**
C19orf59	589.22	1742.89	0.0518	
CAMKK2	258.21	344.56	0.0455	*
CARD17	73.59	313.96	0.0009	***
CASP5	276.61	922.42	0.0028	**
CATSPER1	22.63	26.05	0.5671	
CDK5RAP2	44.65	93.88	0.0377	*
CEACAM1	247.57	413.94	0.0841	
CEACAM3	51.03	87.26	0.0234	*
CKAP4	1219.77	2068.49	0.1619	
CLC	1143.97	559.53	0.0855	
COL4A3BP	459.13	544.99	0.1465	
CR1	2217.03	4296.86	0.0356	*
CSF3R	3770.74	6616.38	0.0140	*
CYP1B1	88.99	345.88	0.0015	**
DHRS13	178.48	378.62	0.0248	*
DKFZp761E198	465.20	691.61	0.0249	*
DOK3	130.87	237.99	0.0103	*
DSC2	300.85	589.41	0.0167	*
ECE1	1344.25	2324.59	0.0025	**
EEF1D	1785.44	1624.26	0.2707	
ELF2	557.32	693.76	0.0306	*
ERI1	73.00	108.53	0.0670	
FCGR1A	592.15	1026.34	0.0228	*
FCGR1C	4703.10	8674.45	0.0135	*
FCRL2	45.63	42.54	0.8376	
FLJ35801	7.74	7.65	0.9323	
FLJ43093	1.46	2.02	0.2699	
FUT6	4.24	5.42	0.1511	
GK	705.24	1409.77	0.0167	*
GLT1D1	949.49	1895.28	0.0002	***
GNB4	273.33	379.38	0.0011	**
GPR141	64.82	89.93	0.1949	
GPR160	682.25	1209.05	0.0173	*
GPR175	25.15	26.94	0.5159	
GPR97	266.44	525.37	0.0191	*
H2AFJ	15.47	17.15	0.3521	
HECW2	42.18	71.65	0.0060	**
HIST1H2BD	2941.88	3346.64	0.3558	
HIST1H4H	1636.12	1512.92	0.5923	
HIST2H2AB	493.41	518.08	0.7857	
HIST2H2AC	2206.40	1890.96	0.1203	
IFIT3	573.02	849.42	0.0321	*
IFITM3	6737.24	15779.60	0.0006	***
IGSF6	1972.23	3053.19	0.0069	**
IL18R1	961.56	1887.02	0.1460	
IL1R2	653.16	1565.68	0.1590	
KCNJ15	1160.90	2264.36	0.0026	**
KIAA0232	528.80	582.17	0.1545	
KLHL2	543.12	1068.11	0.0026	**
LILRA5	1906.61	4243.82	0.0037	**
LIMK2	1018.30	1860.16	0.0177	*
LIN7A	250.24	371.81	0.0552	
LOC100128460	11.36	19.93	0.0419	*
LOC100130562	3708.09	2870.47	0.0218	*
LOC100132499	2373.23	1848.08	0.0222	*
LOC100133177	13.56	17.79	0.1885	
LOC100170939	18.18	15.96	0.3558	
LOC642103	1444.62	2764.56	0.0623	
LOC642469	8.05	7.28	0.4330	
LOC642684	90.93	170.24	0.0578	
LOC644852	3.52	4.49	0.2755	
LOC648984	3.84	5.05	0.1010	
LOC653604	857.33	893.50	0.8491	
LOC728519	130.86	84.43	0.0285	*
LRG1	489.91	1228.30	0.0139	*
LRPAP1	43.77	46.87	0.6611	
MAFF	14.70	17.53	0.3971	
MANSC1	303.03	599.07	0.0025	**
MAPK13	402.27	444.14	0.6384	
MAPK14	2266.86	4221.26	0.0093	**
MIAT	313.17	173.30	0.0078	**
MIIP	158.46	180.11	0.3250	
MMP25	672.95	1212.92	0.0093	**
MRPL40	67.22	62.85	0.6462	
NAMPT	13088.60	31368.40	0.0004	***
NBN	1005.20	1575.33	0.0538	
NLRC4	207.13	418.41	0.0230	*
OAS1	1477.21	2168.77	0.0459	*
PAG1	730.98	1061.77	0.0642	
PFKFB3	1013.04	1966.64	0.0446	*
PGS1	257.69	413.91	0.0348	*
PHAX	106.41	95.82	0.5238	
PPP2R3A	5.84	6.65	0.5077	
PRDX6	553.33	585.15	0.5877	
PSG9	4.47	7.84	0.0050	**
RAB20	228.42	463.00	0.0239	*
RASGRP4	645.30	983.33	0.0263	*
RBM47	339.57	631.79	0.0027	**
RHOBTB1	58.73	88.72	0.0346	*
RP2	453.97	692.84	0.0009	***
SEMA6B	7.14	10.98	0.3176	
SERPINA1	2152.24	3556.25	0.0089	**
SERPING1	1235.93	2339.85	0.0138	*
SIGLEC9	190.09	380.09	0.0114	*
SIPA1L2	13.68	29.53	0.0057	**
SMARCD3	60.36	130.47	0.0092	**
SPRYD3	201.40	216.00	0.4472	
SPTY2D1	108.79	123.68	0.2023	
TDRD9	65.16	73.19	0.8162	
TLR10	91.85	163.07	0.0033	**
TLR2	1152.99	2622.20	0.0008	***
TLR4	1444.44	2942.07	0.0012	**
TLR5	108.19	223.30	0.0071	**
TP53I3	25.24	35.06	0.2268	
TPST1	66.79	172.59	0.0169	*
TRIB1	219.94	409.18	0.0041	**
TUFT1	12.96	21.06	0.0193	*
VNN1	1081.94	2472.72	0.1388	
ZAK	12.04	28.25	0.0005	***
ZBTB16	54.95	220.63	0.0701	

In a technically independent setup using probes with sequences different from the microarray, all but one of the 125 differentially abundant transcripts identified by the microarray at week 2 were validated by NanoString (see also Figure 3b). The *FLJ20273* gene was excluded due to poor probe specificity. Seventy-six transcripts were found to be significantly differentially abundant in TB-IRIS using MW*U* test with BH-FDR. * $p \leq 0.05$, ** $p \leq 0.01$, *** $p \leq 0.001$
